# AI- and Security-Empowered End–Edge–Cloud Modular Platform in Complex Industrial Processes: A Case Study on Municipal Solid Waste Incineration

**DOI:** 10.3390/s25226973

**Published:** 2025-11-14

**Authors:** Jian Tang, Tianzheng Wang, Hao Tian, Wen Yu

**Affiliations:** 1 School of Information Science and Technology, Beijing University of Technology, Beijing 100124, China; wangtz@emails.bjut.edu.cn (T.W.); tianh@emails.bjut.edu.cn (H.T.); 2 Beijing Laboratory of Smart Environmental Protection, Beijing 100124, China; 3 Departamento de Control Automatico, CINVESTAV-IPN (National Polytechnic Institute), Mexico City 07360, Mexico; wen.yu@cinvestav.mx

**Keywords:** complex industrial process, operation optimization control, AI security-empowered end–edge–cloud collaboration application, hardware-in-loop platform, municipal waste solid incineration

## Abstract

Achieving long-term stable optimization in complex industrial processes (CIPs) is notoriously challenging due to their unclear physical/chemical reaction mechanisms, fluctuating operating conditions, and stringent regulatory constraints. A significant gap persists between promising artificial intelligence (AI) algorithms developed in academic research and their practical deployment in industrial actual processes. To bridge this gap, this article introduces the AI- and security-empowered end–edge–cloud modular platform (AISE^3^CMP). It consists of four systems such as whole-process AI modeling, end-side basic loop and AI-assisted decision-making, edge-side security isolation and AI control, and cloud-side security transmission and AI optimization. The data isolation collection module of the platform was deployed at a municipal solid waste incineration (MSWI) power plant in Beijing, where it collected multimodal data from real-world industrial sites. The platform’s functionality and effectiveness were validated through the software and hardware developed at the Smart Environmental Protection Beijing Laboratory. The experimental results show efficient and reliable signal transmission between the systems, confirming the platform’s ability to meet the computational demands of AI-based optimization and control algorithms. Compared to previous platforms, AISE^3^CMP features a dual-security transmission mechanism to mitigate data exchange risks and a modular design to enhance integration efficiency. To the best of our knowledge, this platform is the first prototype of a portable, end-to-end cloud platform with a dual-layer security mechanism for CIPs. While the platform effectively addresses data transmission security, further strengthening of cloud-side data protection and ensuring operational safety on the end-side remain significant challenges for the future. Additionally, utilizing this architecture to enable multi-region and multi-plant data sharing, in order to develop industry-specific large language models, represents a key research direction.

## 1. Introduction

Complex industrial processes (CIPs) are pillar industries that support national economic stability and sustainable resource use [1]. Their operational goal is to maximize quality and efficiency, minimize resource consumption, and ensure compliance with environmental standards within specified ranges [2]. The traditional production mode—marked by high energy use, heavy pollutant emissions, and expert-driven manual control and fault diagnosis [3,4]—no longer meets the demands of global competition, tightening low-carbon requirements, or the move toward intelligent, sustainable development enabled by end–edge–cloud collaboration [5,6]. Consequently, adopting innovative technologies and accelerating digital transformation have become inevitable for development [7].

The rapid advancement of artificial intelligence (AI) provides effective tools for intelligent optimization, control, and modeling of CIPs [8]. These AI techniques include particle swarm optimization (PSO) [9], decision trees [10], neural network controllers [11,12], and fuzzy neural networks (FNNs) [13], along with their corresponding improved versions. However, many CIPs operate under harsh conditions—high temperature, high pressure, and toxic gas emissions—which introduce significant uncertainty and safety risks for direct AI deployment [14]. Additionally, the closed nature of on-site control systems, limited computing resources, and the risks of virus or hacker intrusion into control networks present major challenges for the AI application [15]. In this context, engineering validation of AI algorithms in CIPs is required to bridge research development and industrial process deployment.

To enable such validation, some studies adopt fully virtualized simulation platforms that complete AI algorithm testing entirely in simulation. For instance, Sun et al. [16] proposed a real-time electromagnetic transient simulation platform based on graphics processing units (GPUs), achieving higher scalability and reduced computational costs. Similarly, the subsequent developments in power system simulation software have improved power flow solutions and enhanced dynamic simulation performance [17]. In the field of large-scale coal preparation, Dai et al. [18] integrated model predictive control with reinforcement learning within a simulation platform to investigate multi-rate optimization of ash content. In the field of power systems, Khurram et al. [19] developed a platform for real-time collaboration between grids and distributed energy resources. Compared with verification workflows based on MATLAB, Python, and other tools, these platforms separate the control system and the controlled process into modules and simulate data exchange through interfaces with explicit physical meaning. Nevertheless, most fully virtualized simulation platforms overlook noise, interference, and other real-world uncertainties, which limits the practical deployment of AI techniques.

To address the limitations of the aforementioned platforms, researchers have begun incorporating actual control systems into simulation environments. This integration, typically involving multiple computers, industrial controllers, digital signal conversion boards, and other physical devices, has led to the development of hardware-in-the-loop simulation platforms (HILSPs) for CIPs [20,21]. For example, Rankin et al. [22] developed a platform for nuclear power plants to verify safety control systems. Xie et al. [23] designed a megawatt-level, three-bladed horizontal wind turbine platform capable of simulating both static and dynamic characteristics across the full speed range, proving its suitability for industrial production and laboratory research. Debnath et al. [24] validated the layered control systems in solar power plants using multiple HILSPs, with results confirming continuous operation even under fault conditions. Guan et al. [25] built a HILSP to study integrated thermoelectric energy systems, offering a low-cost and precise tool. In power systems and distribution networks, Mao et al. [26] established a HILSP for three-phase four-wire power systems to ensure grid safety, stability, and optimized operation. Similarly, Cheng et al. [27] developed a HILSP for microgrids to model and study intelligent electronic devices in the power system. These efforts enhance the authenticity and reliability of simulation platforms and provide a validation environment that closely matches real-world conditions. Evidently, HILSPs bridge the gap between laboratory development and field deployment. They preserve repeatability and manageable risk, enabling further engineering validation and practical deployment of AI algorithms in CIPs such as the MSWI process [8].

Despite their benefits, HILSPs still face challenges in CIP scenarios. To improve real–virtual interoperability, recent studies adopt standardized communication protocols and event-driven synchronization, which address communication and timing issues [28,29,30,31]. At the same time, limited hardware and computing capacity constrains real-time simulation and large-scale operational optimization; a practical remedy is to augment HILSPs with cloud-side and edge-side computing [32,33,34,35]. However, two practical aspects remain under-addressed: the relative independence of existing control systems in CIP plants and the need for emergency protocols to ensure safe operation under critical conditions. More importantly, the gradual acceptance of AI by domain experts is itself a security concern: without trusted workflows and safeguards, practical deployment remains elusive. While several studies employ AI techniques to mitigate these risks [36,37], they did not fundamentally eliminate security vulnerabilities through physical isolation. Therefore, how to further strengthen security protection remains a key problem that urgently needs to be addressed.

To this end, this article proposes the AI security-empowered end–edge–cloud modular platform (AISE^3^CMP) for CIPs. The innovations are as follows: (1) A dual-layer physically isolated secure data transmission mechanism is proposed, ensuring undisturbed forward data collection, reliable data transmission, and secure reverse data transmission. (2) A modular and portable architecture is proposed, offering flexible and configurable functions. (3) A novel definition of end-side control systems for CIP based on PLC/DCS is proposed, along with end-side basic loop and AI-assisted decision-making strategies, ensuring the practical deployment of AI security empowerment (AI and security empowerment: Invoke AI capabilities within strictly controlled security boundaries while enforcing physical and logical isolation along the transfer paths of both collected data and AI-generated outputs). (4) A targeted municipal solid waste incineration (MSWI) simulation platform, AISE^3^CMP-MSWI, has been developed. It verifies the effectiveness of data transmission in the proposed design and, through representative cases, demonstrates the efficacy of the modular architecture. 

The rest of this article is as follows. Section 2 gives the abstractly current stratus and requirements in terms of the operational optimization of CIPs. Section 3 describes the functional design of the proposed AISE^3^CMP. Section 4 adopts the municipal waste solid incineration (MSWI) process as a typical case to build the AISE^3^CMP-MSWI and verify it. Section 5 provides a summary and outlook.

## 2. Description of Operational Optimization Problems in CIP

### 2.1. Description of Current Status in CIPs

**Figure 1:**
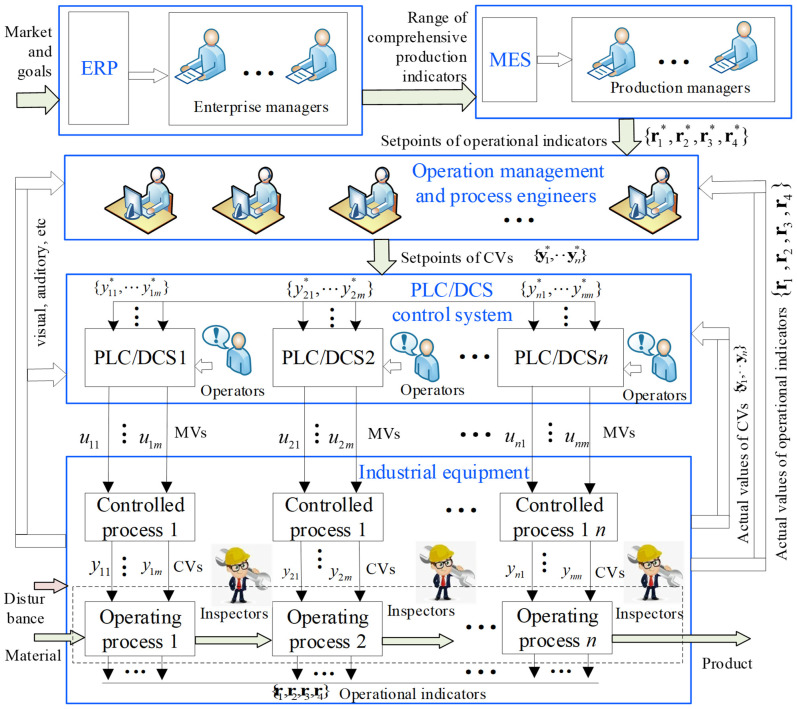
Operation process of CIPs.

In the operation process of CIPs, domain experts—managers, engineers, and operators—leverage long-term practice to combine perception, cognition, and operation, achieving operational optimization through human–machine [2], as shown in Figure 1.

As shown in Figure 1, domain experts in different positions adopt embodied intelligence to achieve operational optimization of CIPs. Managers first use ERP/MES and external information to set quality, efficiency, consumption, and environmental indicators $\left\{r_{1}^{*},r_{2}^{*},r_{3}^{*},r_{4}^{*}\right\}$ and make informed decisions. Operation management and process engineers then determine setpoints and acceptable ranges for controlled variables (CVs) $\left\{y_{1}^{*},\ldots ,y_{n}^{*}\right\}$ based on these targets and on laboratory/online measurements and multimodal production data (video, audio, inspection, etc.). Finally, the PLC/DCS tracks the CVs in automatic or manual mode, generates the manipulated variable (MV) vector, and drives the process to keep indicators within range.

### 2.2. Description of Requirements for Operational Optimization in CIP

The goals of CIP operation are to maintain operational indicators within the ranges while ensuring production safety,

(1) $$r_{j}^{\min}\leq r_{j}\left(t\right)\leq r_{j}^{\max},\;j=1,2,3,4$$

maximize product quality and efficiency indicators,

(2) $$\max r_{1}\left(t\right),r_{2}\left(t\right)$$

and minimize consumption indicators such as material and energy consumption, as well as pollutant emission concentrations (environmental indicators) as much as possible,

(3) $$\min r_{3}\left(t\right),r_{4}\left(t\right)$$

Given these objectives, long-term operation must determine the optimal operational indices under above multiple factors; this can be expressed as follows:

(4) $$J_{1}(t)=f_{1}\left(\max\left(r_{1}\left(t\right),r_{2}\left(t\right)\right);\min\left(r_{3}\left(t\right),r_{4}\left(t\right)\right)\right)$$

Then, the controller tracks the CV setpoints under amplitude and rate limits,

(5) $$\begin{array}{c} \min\begin{array}{c} J_{1}(k)=\frac{1}{T}\sum_{t=0}^{T}{\left(y_{nm}\left(t\right)-y_{nm}^{*}\left(t\right)\right)}^{2} \end{array}\\ s.t.\left\{\begin{array}{c} y_{nm}^{\min}\leq y_{nm}\left(t\right)\leq y_{nm}^{\max}\\ \Delta y_{nm}^{\min}\leq \left|y_{nm}\left(t\right)-y_{nm}\left(t-1\right)\right|\leq \Delta y_{nm}^{\max}\\ y_{nm}\left(t\right)=f_{nm}^{\mathrm{Controlled}}\left(y_{nm}\left(t\right),u_{nm}\left(t\right),d_{nm}^{y}\left(t\right)\right)\\ u_{nm}^{\min}\leq u_{nm}\left(t\right)\leq u_{nm}^{\max}\\ \Delta u_{nm}^{\min}\leq \left|u_{nm}\left(t\right)-u_{nm}\left(t-1\right)\right|\leq \Delta u_{nm}^{\max} \end{array}\right. \end{array}$$

Obviously, manual modes cannot adequately accomplish the above tasks; AI assistance becomes essential. Yet, the application of AI algorithms in CIPs faces two practical issues:

(a) On-site control. Controllers must compute feasible MVs under strict latency and safety constraints. Edge-side computing is, therefore, required to supplement limited on-site resources and to guarantee secure forward data acquisition and secure reverse/control backhaul.
(b) Operational-index optimization. Unlike end-side loops with modest demand, determining optimal operating conditions under multi-factor coupling requires substantially more data and computation. This calls for cloud-side resources and scalable data management.

## 3. Structure and Function of AISE^3^CMP

**Figure 2:**
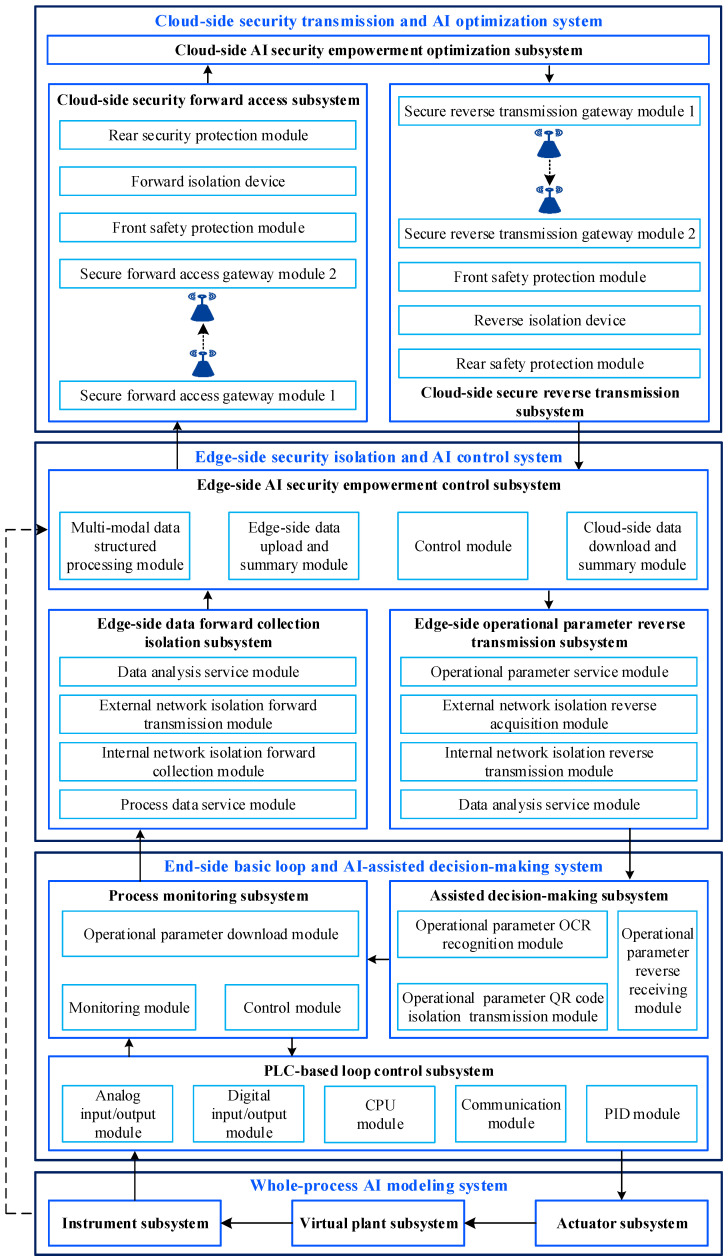
Structure of AISE^3^CMP.

The structure of the AISE^3^CMP is illustrated in Figure 2. 

The function brief description of different system is as follows:

(1) Whole-process AI modeling system. It integrates actual physical devices with AI algorithms to accurately simulate actuators, plants, and instrumentation devices. It can replicate operational processes and offer data support functions to ensure closed-loop control.
(2) End-side basic loop and AI-assisted decision-making system. It utilizes actual control devices to perform the basic loop control tasks. Simultaneously, the system incorporates AI-assisted decision-making functions to provide real-time decisions by analyzing the upper-level operational parameters.
(3) Edge-side security isolation and AI control system. It employs physical security isolation devices to facilitate bidirectional data isolation transmission between the end side and the edge side. Additionally, it provides the capability to upload multi-modal data and load operational parameters. More importantly, it can leverage AI control algorithms to implement control based on multi-modal data.
(4) Cloud-side security transmission and AI optimization system. It utilizes wireless security devices to ensure secure data transmission between the edge side and the cloud side, thereby enabling the optimal solution for CIP operations based on AI algorithms.

**Remark** **1.** *This article proposes a platform architecture designed to bridge the gap between theoretical research and practical deployment through the integration of AI and various hardware/software components. In doing so, we provide a universal framework from the perspective of AI algorithms, such as those required for modeling, control, and optimization in CPS research. Throughout the article, the term "AI algorithm" refers to a category of methods, rather than a single one. To clearly present inputs and outputs while keeping the discussion accessible, we adopt simplified formulations in the main text. Specifically, this encompasses AI techniques developed to solve problems in CPS. For clarity, we briefly describe the AI algorithms used in the MSWI process. These include interpretable decision tree algorithms and fuzzy neural network algorithms for constructing AI-based controlled objects models, deep neural network algorithms for AI-based environmental indicator models, convolutional neural network algorithms for AI-based image recognition, neural network control and model predictive control algorithms for AI-based control, and particle swarm optimization (PSO) for AI-based pollution emissions optimization*.

### 3.1. Whole-Process AI Modeling System

#### 3.1.1. Actuator Subsystems

This subsystem simulates actuators in CIP, including pumps, valves, and other devices. Upon receiving the standard industrial electrical signals transmitted by the PLC-based loop control subsystem through hardware, the established actuator model converts them into actual values with physical meaning. The input and output are as follows:

(6) $$u_{m}^{\mathrm{Act}}=f_{m}^{A}\left(u_{m}^{\mathrm{Ele}},P_{m,\max}^{A},P_{m,\min}^{A},U_{m,\max}^{A},U_{m,\min}^{A}\right)$$

where m=1,2,…,M. $u_{m}^{\mathrm{Act}}$ and $u_{m}^{\mathrm{Ele}}$ represent the output and the electrical signal value of the *m*-th actuator variables, respectively; $P_{m,\max}^{A}$ and $P_{m,\min}^{A}$ represent the upper and lower limits of the *m*-th actuator variables, respectively; $U_{m,\max}^{A}$ and $U_{m,\min}^{A}$ represent the upper and lower limits of the standard industrial electrical signal values, respectively; and $f_{m}^{A}\left(∙\right)$ represents the *m*-th established actuator model, which consists of a range conversion model and a simulation model with serial connection.

The range conversion model $g_{m}^{A}\left(∙\right)$ can be represented as follows:

(7) $$u_{m}^{\mathrm{Ana}}=\frac{P_{m,\max}^{A}-P_{m,\min}^{A}}{U_{m,\max}^{A}-U_{m,\min}^{A}}u_{m}^{\mathrm{Ele}}$$

where $u_{m}^{\mathrm{Ana}}$ is the output of the range conversion model. 

Additionally, the simulation model $h_{m}^{A}\left(∙\right)$ can be simulated using proportional, integral, differential, and first-order inertial components, as follows:

(8) $$u_{m}^{\mathrm{Act}}\left(t\right)=Ku_{m}^{\mathrm{Ana}}\left(t\right)$$

(9) $$u_{m}^{\mathrm{Act}}\left(t\right)=u_{m}^{\mathrm{Act}}\left(t-1\right)+Ku_{m}^{\mathrm{Ana}}\left(t\right)$$

(10) $$u_{m}^{\mathrm{Act}}\left(t\right)=K\left(u_{m}^{\mathrm{Ana}}\left(t\right)-u_{m}^{\mathrm{Ana}}\left(t-1\right)\right)$$

(11) $$u_{m}^{\mathrm{Act}}\left(t\right)=\left(1-\alpha \right)u_{m}^{\mathrm{Act}}\left(t-1\right)+K\alpha u_{m}^{\mathrm{Ana}}\left(t\right)$$

where K represents system gain, and α represents the response time coefficient.

#### 3.1.2. Virtual Plant Subsystem

This subsystem simulates the CIP process, providing feedback values for the upper-level system. The proposed AISE^3^CMP employs AI algorithms to establish a virtual plant model based on industrial big data, as follows:

(12) $$\left[y_{1}^{\mathrm{Act}},y_{2}^{\mathrm{Act}},\cdot \cdot \cdot ,y_{Q}^{\mathrm{Act}}\right]=f_{\mathrm{AI}}^{\mathrm{Plant}}\left(z_{1},z_{2},\cdot \cdot \cdot ,z_{P}\right)$$

where $\left[y_{1}^{\mathrm{Act}},y_{2}^{\mathrm{Act}},\cdot \cdot \cdot ,y_{Q}^{\mathrm{Act}}\right]$ represents the output of the virtual plant model, which is the required detection variables for the instrument devices; $\left[z_{1},z_{2},\cdot \cdot \cdot ,z_{P}\right]$ represents the input of the model, which includes a combination of partial actuator variables $\left[u_{1}^{\mathrm{Act}},u_{2}^{\mathrm{Act}},\cdot \cdot \cdot ,u_{M}^{\mathrm{Act}}\right]$ and instrument device variables $\left[y_{1}^{\mathrm{Act}},y_{2}^{\mathrm{Act}},\cdot \cdot \cdot ,y_{Q}^{\mathrm{Act}}\right]$ with P≤M+Q.

To improve the accuracy of the constructed virtual plant model and align it with the previously described the operational optimization problem in CIP, this article further divides the virtual plant model into a controlled process model and an operational indicator model:

(13) $$f_{\mathrm{AI}}^{\mathrm{Plant}}\left(Z\right)=f_{\mathrm{AI}}^{\mathrm{Controlled}}\left(Z_{1}\right)\circ f_{\mathrm{AI}}^{\mathrm{Operational}}\left(Z_{2}\right)$$

(14) $$\left[y_{1}^{\mathrm{Controlled}},y_{2}^{\mathrm{Controlled}},\cdot \cdot \cdot ,y_{Q_{1}}^{\mathrm{Controlled}}\right]=f_{\mathrm{AI}}^{\mathrm{Controlled}}\left(z_{1},z_{2},\cdot \cdot \cdot ,z_{P_{1}}\right)$$

(15) $$\left[y_{1}^{\mathrm{Index}},y_{2}^{\mathrm{Index}},\cdot \cdot \cdot ,y_{Q_{2}}^{\mathrm{Index}}\right]=f_{\mathrm{AI}}^{\mathrm{Operational}}\left(z_{1},z_{2},\cdot \cdot \cdot ,z_{P_{2}}\right)$$

The outputs of above models are merged as $\left[y_{1}^{\mathrm{Act}},y_{2}^{\mathrm{Act}},\cdot \cdot \cdot ,y_{Q}^{\mathrm{Act}}\right]$ and denoted as $R^{\mathrm{Act}}$. Thus, there exist $\left[y_{1}^{\mathrm{Controlled}},\cdot \cdot \cdot ,y_{Q_{1}}^{\mathrm{Controlled}}\right]\subseteq R^{\mathrm{Act}}$ and $\left[y_{1}^{\mathrm{Index}},\cdot \cdot \cdot ,y_{Q_{2}}^{\mathrm{Index}}\right]\subseteq R^{\mathrm{Act}}$ with $Q_{1}+Q_{2}=Q$.

#### 3.1.3. Instrument Subsystem

This subsystem simulates sensors and measurement devices in CIP by integrating AI algorithms with hardware. Upon receiving the outputs from the virtual plant subsystem via OPC, the established instrument model converts into electrical form and transmits them to the PLC-based loop control subsystem. The corresponding inputs and outputs are summarized as follows:

(16) $$y_{q}^{\mathrm{Ele}}=f_{q}^{I}(y_{q}^{\mathrm{Act}},P_{q,\max}^{I},P_{q,\min}^{I},U_{q,\max}^{I},U_{q,\min}^{I})$$

where q=1,2,…,Q. $y_{q}^{\mathrm{Ele}}$ represents the output of the *q*-th instrument model; $P_{q,\max}^{I}$ and $P_{q,\min}^{I}$ represent the upper and lower limits of the *q*-th physical values, respectively; $U_{q,\max}^{I}$ and $U_{q,\min}^{I}$ represent the upper and lower limits of the standard industrial electrical signal values, respectively; and $f_{q}^{I}\left(∙\right)$ represents the *q*-th instrument model, consisting of a simulation model and a range conversion model $g_{i}^{I}\left(∙\right)$ with serial connection.

The simulation model $h_{i}^{I}\left(∙\right)$ can also be simulated using proportional, integral, derivative, and first-order inertial elements, and its range conversion model $g_{i}^{I}\left(∙\right)$ can be represented as follows:

(17) $$y_{q}^{\mathrm{Ele}}=\frac{U_{q,\max}^{I}-U_{q,\min}^{I}}{P_{q,\max}^{I}-P_{q,\min}^{I}}y_{q}^{\mathrm{Ana}}$$

where $y_{i}^{\mathrm{Ana}}$ is the output of the range conversion model.

**Remark** **2.** *AI algorithms can be employed to develop data-driven models based on actuator and instrument device data, effectively replacing the range conversion models and simulation models*.

### 3.2. End-Side Basic Loop and AI-Assisted Decision-Making System

#### 3.2.1. Assisted Decision-Making Subsystem

This subsystem retrieves the operational parameters from the edge-side operational parameter reverse transmission subsystem via the operational parameter reverse receiving module and transmits them to the process monitoring subsystem. Its input and output are as follows:

(18) $$\left[D_{\mathrm{ADS}}^{\mathrm{End}}\right]=f_{\mathrm{ADS}}^{\mathrm{End}}\left(D_{\mathrm{EOPRTS}}^{\mathrm{Edge}},{†}_{\mathrm{ADS}}^{\mathrm{End}}\right)$$

where $D_{\mathrm{ADS}}^{\mathrm{End}}$ represents the operational parameters after decision-making with the operator; $D_{\mathrm{EOPRTS}}^{\mathrm{Edge}}$ represents the operational parameters transmitted by the edge-side operational parameters reverse transmission subsystem, which can be denoted as

(19) $$D_{\mathrm{EOPRTS}}^{\mathrm{Edge}}=\left[u_{\mathrm{EOPRTS}}^{\mathrm{Edge}},K_{\mathrm{EOPRTS}}^{\mathrm{Edge}},r_{\mathrm{CSRTS}}^{\mathrm{Cloud}},p_{\mathrm{CSRTS}}^{\mathrm{Cloud}}\right]$$

where $u_{\mathrm{EOPRTS}}^{\mathrm{Edge}}$ and $K_{\mathrm{EOPRTS}}^{\mathrm{Edge}}$ are the MV values and the PID parameters solved by AI algorithms in the edge-side security isolation and AI control system, $r_{\mathrm{CSRTS}}^{\mathrm{Cloud}}$ and $p_{\mathrm{CSRTS}}^{\mathrm{Cloud}}$ are the optimal setpoint values and prediction values of key variables solved by AI algorithms in the cloud-side security transmission and optimization system; ${†}_{\mathrm{ADS}}^{\mathrm{End}}$ represents the transmission method for operational parameters, which can use any of the following three methods: direct transmission, OCR recognition, and QR code isolation transmission. Among them, OCR recognition requires a camera device and text recognition algorithm. QR code isolation transmission simulates human eye recognition and utilizes color QR code encoding and decoding to establish a one-way isolation channel based on a non-contact, one-way import device.

**Remark** **3.** *To further ensure the secure isolation of CIP plants and guarantee safe operation and production, during the initial phase of AI algorithm verification, the subsystem can utilize OCR recognition and QR code isolation transmission to display operational parameters on the front-end interface for reference by domain experts. Once the AI results are accepted by operators, these operational parameters can be directly transmitted to the process monitoring subsystem with OPC*.

#### 3.2.2. Process Monitoring Subsystem

This subsystem is supported by control configuration software from PLC/DCS manufacturers to download operational parameters. While monitoring the whole-process AI modeling system, it also controls the virtual CIP process based on the specified operational parameters. The input–output relationship is as follows:

(20) $$\left[r_{\mathrm{PMS}}^{\mathrm{End}},u_{\mathrm{PMS}}^{\mathrm{End}},K_{\mathrm{PMS}}^{\mathrm{End}}\right]=f_{\mathrm{PMS}}^{\mathrm{End}}\left(u^{\mathrm{Ana}},y^{\mathrm{Ana}},D_{\mathrm{ADS}}^{\mathrm{End}},{†}_{\mathrm{PMS}}^{\mathrm{End}}\right)$$

where $r_{\mathrm{PMS}}^{\mathrm{End}}$, $u_{\mathrm{PMS}}^{\mathrm{End}}$, and $K_{\mathrm{PMS}}^{\mathrm{End}}$ represent the CV setpoint values, MV values, and PID parameters sent from the process monitoring subsystem to the PLC-based loop control subsystem, respectively; and ${†}_{\mathrm{PMS}}^{\mathrm{End}}$ represents the control mode. Considering the control strategies employed by operators in actual industrial sites, two control modes can be chosen: PID module based on setpoint tracking control and directly download MV values based on operators.

#### 3.2.3. PLC-Based Loop Control Subsystem

The actual control system is employed to facilitate signal conversion and loop control between the process monitoring subsystem and the whole-process AI modeling system. The signal conversion process mirrors the range conversion model utilized in both the actuator and the instrument; thus, it will not be reiterated here. The input–output relationship of the loop control process is as follows:

(21) $$u_{m}^{\mathrm{Ele}}=f_{\mathrm{PLC},\mathrm{Control},m}^{\mathrm{End}}\left(r_{\mathrm{PMS},m}^{\mathrm{End}},u_{\mathrm{PMS},m}^{\mathrm{End}},u^{\mathrm{Ana}},y^{\mathrm{Ana}},C_{m}^{A},{†}_{\mathrm{PMS},m}^{\mathrm{End}}\right)$$

where $C_{m}^{A}=\left[P_{m,\max}^{A},P_{m,\min}^{A},U_{m,\max}^{A},U_{m,\min}^{A}\right]$; $f_{\mathrm{PLC},\mathrm{Control},m}^{\mathrm{End}}\left(∙\right)$ and ${†}_{\mathrm{PMS},m}^{\mathrm{End}}$ represent the *m*-th loop control process and its control mode. If the MV values are directly downloaded, the loop calculation will be omitted. On the contrary, the incremental algorithm is as follows:

(22) $$\begin{array}{c} u_{\mathrm{PMS},\mathrm{PLC},m}^{\mathrm{End}}\left(k\right)=u_{\mathrm{PMS},\mathrm{PLC},m}^{\mathrm{End}}\left(k-1\right)+K_{P,m}^{\mathrm{End}}\left[e_{\mathrm{PMS},m}^{\mathrm{End}}\left(k\right)-e_{\mathrm{PMS},m}^{\mathrm{End}}\left(k-1\right)\right]+K_{I,m}^{\mathrm{End}}e_{\mathrm{PMS},m}^{\mathrm{End}}\left(k\right)+\\ K_{D,m}^{\mathrm{End}}\left[e_{\mathrm{PMS},m}^{\mathrm{End}}\left(k\right)-2e_{\mathrm{PMS},m}^{\mathrm{End}}\left(k-1\right)+e_{\mathrm{PMS},m}^{\mathrm{End}}\left(k-2\right)\right] \end{array}$$

where $u_{\mathrm{PMS},\mathrm{PLC},m}^{\mathrm{End}}$ and $e_{\mathrm{PMS},m}^{\mathrm{End}}$ represent the MV value and error, respectively; $K_{P,m}^{\mathrm{End}}$, $K_{I,m}^{\mathrm{End}}$, and $K_{D,m}^{\mathrm{End}}$ represent the proportional, integral, and derivative coefficients of the *m*-th PID loop.

### 3.3. Edge-Side Security Isolation and AI Control System

#### 3.3.1. Edge-Side Data Forward Collection Isolation Subsystem

This subsystem acquires process data from the process monitoring subsystem and implements forward-only transmission to the external network. The workflow is as follows: (1) The process data service module collects process data from the process monitoring subsystem. The internal network isolation forward collection module then sends the them and the associated acquisition configuration files to the external network over unidirectional optical fiber. (2) The external network isolation forward transmission module receives the process data and configuration files. (3) The data analysis service module consolidates and analyzes the received process data and configuration files and provides data services to edge-side devices via the OPC protocol; this can be represented as follows:

(23) $$D_{\mathrm{EDFCIS}}^{\mathrm{Edge}}=f_{\mathrm{EDFCIS}}^{\mathrm{Edge}}\left(D_{\mathrm{PMS}}^{\mathrm{End}},S_{\mathrm{EDFCIS}}^{\mathrm{Edge}},T_{\mathrm{EDFCIS}}^{\mathrm{Edge}},IP_{\mathrm{Acq},\mathrm{In}}^{\mathrm{Edge}},IP_{\mathrm{Acq},\mathrm{Out}}^{\mathrm{Edge}},{†}_{\mathrm{EDFCIS}}^{\mathrm{Edge}}\right)$$

where $D_{\mathrm{EDFCIS}}^{\mathrm{Edge}}$ and $D_{\mathrm{PMS}}^{\mathrm{End}}$ represent the process data published by the edge-side data forward collection isolation subsystem and the process data running in the process monitoring subsystem, respectively; $S_{\mathrm{EDFCIS}}^{\mathrm{Edge}}$ and $T_{\mathrm{EDFCIS}}^{\mathrm{Edge}}$ represent a set of data tags and the time interval for data forward collection; $IP_{\mathrm{Acq},\mathrm{In}}^{\mathrm{Edge}}$ and $IP_{\mathrm{Acq},\mathrm{Out}}^{\mathrm{Edge}}$ represent the IP addresses of the internal and external network, respectively; and ${†}_{\mathrm{EDFCIS}}^{\mathrm{Edge}}$ represents the data transmission mode, which is only readable here.

#### 3.3.2. Edge-Side AI Security Empowerment Control Subsystem

This subsystem preprocesses the collected multi-modal data and transmits it to the cloud-side security forward access subsystem. Simultaneously, it downloads the operational parameters from the cloud-side secure reverse transmission subsystem to the edge-side operational parameter reverse transmission subsystem. This process can be represented as follows:

(24) $$\left[D_{\mathrm{EAISECS},\mathrm{Up}}^{\mathrm{Edge}},D_{\mathrm{EAISECS},\mathrm{Down}}^{\mathrm{Edge}}\right]=f_{\mathrm{EAISECS}}^{\mathrm{Edge}}\left(D_{\mathrm{EDFCIS}}^{\mathrm{Edge}},D_{\mathrm{Unstructured}},D_{\mathrm{CSRTS}}^{\mathrm{Cloud}}\right)$$

where $D_{\mathrm{EDFCIS}}^{\mathrm{Edge}}$, $D_{\mathrm{Unstructured}}$, and $D_{\mathrm{CSRTS}}^{\mathrm{Cloud}}$ represent the process data published by the edge-side data forward collection isolation subsystem, the unstructured data of the whole-process AI modeling system, and the operational parameters of the cloud-side secure reverse transmission subsystem, respectively; $D_{\mathrm{EAISECS},\mathrm{Up}}^{\mathrm{Edge}}$ and $D_{\mathrm{EAISECS},\mathrm{Down}}^{\mathrm{Edge}}$ represent the data uploaded from the edge-side AI security empowerment control subsystem to the cloud-side security forward access subsystem and downloaded to the edge-side operational parameter reverse transmission subsystem, respectively. 

We have $D_{\mathrm{EAISECS},\mathrm{Up}}^{\mathrm{Edge}}=\left[D_{\mathrm{EDFCIS}}^{\mathrm{Edge}},D_{\mathrm{Unstructured}}^{\mathrm{Edge}}\right]$, and $D_{\mathrm{Unstructured}}^{\mathrm{Edge}}$ refers to data, such as flame images and text records, that are extracted using AI algorithms. 

Similarly, we have $D_{\mathrm{EAISECS},\mathrm{Down}}^{\mathrm{Edge}}=\left[u_{\mathrm{EAISECS}}^{\mathrm{Edge}},K_{\mathrm{EAISECS}}^{\mathrm{Edge}},r_{\mathrm{CSRTS}}^{\mathrm{Cloud}},p_{\mathrm{CSRTS}}^{\mathrm{Cloud}},\eta_{\mathrm{CSRTS}}^{\mathrm{Cloud}}\right]$, $u_{\mathrm{EAISECS}}^{\mathrm{Edge}}$ and $K_{\mathrm{EAISECS}}^{\mathrm{Edge}}$ are the MV values and optimized PID parameters produced by AI algorithms; $r_{\mathrm{CSRTS}}^{\mathrm{Cloud}}$, $p_{\mathrm{CSRTS}}^{\mathrm{Cloud}}$, and $\eta_{\mathrm{CSRTS}}^{\mathrm{Cloud}}$ are the setpoints, predicted values, and model parameters of key variables transmitted through the cloud-side secure reverse transmission subsystem, that is, $D_{\mathrm{CSRTS}}^{\mathrm{Cloud}}=\left[r_{\mathrm{CSRTS}}^{\mathrm{Cloud}},p_{\mathrm{CSRTS}}^{\mathrm{Cloud}},\eta_{\mathrm{CSRTS}}^{\mathrm{Cloud}}\right]$.

More importantly, the control module in this subsystem has significantly greater computing power. It leverages AI algorithms in combination with multi-modal data to tackle complex control tasks, including PID parameter optimization, MPC control computations, and neural network control calculations. The input–output relationship is as follows:

(25) $$\left[u_{\mathrm{EAISECS}}^{\mathrm{Edge}},K_{\mathrm{EAISECS}}^{\mathrm{Edge}}\right]=f_{\mathrm{AI}}^{\mathrm{Edge}}\left(D_{\mathrm{EAISECS},\mathrm{Up}}^{\mathrm{Edge}},r_{\mathrm{CSRTS}}^{\mathrm{Cloud}},{†}_{\mathrm{EAISECS}}^{\mathrm{Edge}}\right)$$

where ${†}_{\mathrm{EAISECS}}^{\mathrm{Edge}}$ represents the control tasks.

#### 3.3.3. Edge-Side Operational Parameter Reverse Transmission Subsystem

Similar in implementation to the edge-side data forward collection isolation subsystem, this subsystem operates in the reverse direction. It collects operational parameters from the control module and the cloud-side data download and summary module within the edge-side AI security-empowerment control subsystem and delivers them to end-side devices on the local area network via the OPC protocol. This process can be expressed as follows:

(26) $$D_{\mathrm{EOPRTS}}^{\mathrm{Edge}}=f_{\mathrm{EOPRTS}}^{\mathrm{Edge}}\left(D_{\mathrm{EAISECS},\mathrm{Down}}^{\mathrm{Edge}},S_{\mathrm{EOPRTS}}^{\mathrm{Edge}},T_{\mathrm{EOPRTS}}^{\mathrm{Edge}},IP_{\mathrm{Trans},\mathrm{In}}^{\mathrm{Edge}},IP_{\mathrm{Trans},\mathrm{Out}}^{\mathrm{Edge}},{†}_{\mathrm{EOPRTS}}^{\mathrm{Edge}}\right)$$

where $D_{\mathrm{EOPRTS}}^{\mathrm{Edge}}$ represents the operational parameters downloaded to the assisted decision-making subsystem; $S_{\mathrm{EOPRTS}}^{\mathrm{Edge}}$ and $T_{\mathrm{EOPRTS}}^{\mathrm{Edge}}$ represent the tag set and time interval of transmission reverse, respectively; $IP_{\mathrm{Trans},\mathrm{In}}^{\mathrm{Edge}}$ and $IP_{\mathrm{Trans},\mathrm{Out}}^{\mathrm{Edge}}$ represent the IP addresses of the internal and external network, respectively; and ${†}_{\mathrm{EOPRTS}}^{\mathrm{Edge}}$ represents the data transmission mode with only writing.

### 3.4. Cloud-Side Security Transmission and AI Optimization System

#### 3.4.1. Cloud-Side Security Forward Access Subsystem

This subsystem acquires data from the edge side and performs forward-only transmission to the cloud side. The workflow is as follows: (1) The secure forward access gateway modules 1 and 2 use 5G encryption devices to receive wireless data from the edge-side AI security-empowerment control subsystem and apply encryption. (2) The front safety protection module, forward isolation device, and rear security protection module sequentially read the encrypted payloads and enforce industrial-grade security isolation. (3) After controlled decryption and integrity verification, the validated data are released to the cloud-side AI security empowerment optimization subsystem for storage, analytics, and model services. This process can be described as follows:

(27) $$D_{\mathrm{CSFAS}}^{\mathrm{Cloud}}=f_{\mathrm{CSFAS}}^{\mathrm{Cloud}}\left(D_{\mathrm{EAISECS},\mathrm{Up}}^{\mathrm{Edge}},IP_{\mathrm{CSFAS},1}^{\mathrm{Cloud}},IP_{\mathrm{CSFAS},2}^{\mathrm{Cloud}},{†}_{\mathrm{CSFAS}}^{\mathrm{Cloud}}\right)$$

where $IP_{\mathrm{CSFAS},1}^{\mathrm{Cloud}}$ and $IP_{\mathrm{CSFAS},2}^{\mathrm{Cloud}}$ represent the IP addresses of the sender and receiver of the 5G encryption device, respectively; ${†}_{\mathrm{CSFAS}}^{\mathrm{Cloud}}$ represents the encryption method; and $D_{\mathrm{CSFAS}}^{\mathrm{Cloud}}$ represents the multimodal data of the cloud-side security forward access subsystem. This subsystem is solely responsible for encrypting data transmission without performing any secondary processing, thus there exits $D_{\mathrm{CSFAS}}^{\mathrm{Cloud}}\Leftrightarrow D_{\mathrm{EAISECS},\mathrm{Up}}^{\mathrm{Edge}}$.

#### 3.4.2. Cloud-Side AI Security Empowerment Optimization Subsystem

This subsystem retrieves the data from the cloud-side security forward access subsystem and stores it in the database of the cloud server. It carries out time-scale correction, anomaly detection, and missing data filling, providing support for AI modeling, control, optimization, and decision-making calculations at cloud nodes, such as integrating historical and real-time data for online learning and self-updating of prediction models, controlled process models, and operational indicator models, employing intelligent optimization algorithms to determine optimal process parameters suited to current operating conditions, using fault diagnosis algorithms for monitoring current states, future operation forecasting, and state prediction. Finally, the relevant operational parameters are transmission to the cloud-side security reverse transmission subsystem. This process can be expressed as follows:

(28) $$D_{\mathrm{CAISEOS}}^{\mathrm{Cloud}}=f_{\mathrm{CAISEOS}}^{\mathrm{Cloud}}\left(D_{\mathrm{CSFAS}}^{\mathrm{Cloud}},D_{\mathrm{History}}^{\mathrm{Cloud}},D_{\mathrm{CAISEOS}-1}^{\mathrm{Cloud}},{†}_{\mathrm{CAISEOS}}^{\mathrm{Cloud}}\right)$$

where $D_{\mathrm{CAISEOS}}^{\mathrm{Cloud}}$ and $D_{\mathrm{CAISEOS}-1}^{\mathrm{Cloud}}$ represent the operational parameters solved by the cloud-side AI security empowerment optimization subsystem with $D_{\mathrm{CAISEOS}-1}^{\mathrm{Cloud}}\subseteq D_{\mathrm{CAISEOS}}^{\mathrm{Cloud}}$ and $D_{\mathrm{CAISEOS}}^{\mathrm{Cloud}}=\left[r_{\mathrm{CAISEOS}}^{\mathrm{Cloud}},p_{\mathrm{CAISEOS}}^{\mathrm{Cloud}},\eta_{\mathrm{CAISEOS}}^{\mathrm{Cloud}}\right]$; $r_{\mathrm{CAISEOS}}^{\mathrm{Cloud}}$, $p_{\mathrm{CAISEOS}}^{\mathrm{Cloud}}$, and $\eta_{\mathrm{CAISEOS}}^{\mathrm{Cloud}}$ represent the key variable setpoint values, predicted values, and model parameters; and $D_{\mathrm{History}}^{\mathrm{Cloud}}$ represents the historical data stored in the cloud-side data server.

#### 3.4.3. Cloud-Side Secure Reverse Transmission Subsystem

Similar to the cloud-side security forward access subsystem, this subsystem operates in the reverse direction. It encrypts operational parameters generated by the cloud-side AI security empowerment optimization subsystem and delivers them to the edge-side AI security empowerment control subsystem over a reverse-only, security-isolated path; this can be expressed as follows:

(29) $$D_{\mathrm{CSRTS}}^{\mathrm{Cloud}}=f_{\mathrm{CSRTS}}^{\mathrm{Cloud}}\left(D_{\mathrm{CAISEOS}}^{\mathrm{Cloud}},IP_{\mathrm{CSRTS},1}^{\mathrm{Cloud}},IP_{\mathrm{CSRTS},2}^{\mathrm{Cloud}},{†}_{\mathrm{CSRTS}}^{\mathrm{Cloud}}\right)$$

Similarly, this subsystem only is used for encrypted transmission, thus there exits $D_{\mathrm{CSRTS}}^{\mathrm{Cloud}}\Leftrightarrow D_{\mathrm{CAISEOS}}^{\mathrm{Cloud}}$.

Meanwhile, the cloud-side security forward access subsystem and the cloud-side secure reverse transmission subsystem perform encrypted transfer while enforcing physical separation between the edge and the cloud.

**Remark** **4.** *This article focuses on the platform architecture and the establishment of an engineering verification environment. The security architecture is based on the integration of mature isolation and enforcement technologies to ensure controlled data flows. The use of wireless isolation aims to secure data transmission between the factory-side information network and the cloud-side information network outside the factory. Mature market products that comply with local government quarantine regulations are employed for safety isolation*.

### 3.5. Discussion and Analysis

Although end–edge–cloud platforms for CIPs are relatively mature, the proposed AISE^3^CMP differs in four key aspects:

(1) Security for Data Transfer

Forward acquisition and reverse transmit are strictly separated by physical devices, forming dual-layer physical isolation in the end–edge–cloud architecture. Data are exchanged only within independent secure domains, avoiding exposure to nonessential networks and thereby reducing misinjection and unauthorized access. Compared with software-only defenses, source-level physical isolation provides a clearer security boundary and higher reliability, which is well aligned with the stringent stability and compliance requirements of CIP scenarios.

(2) Latency-Oriented Compute Tiering

Compute is orchestrated according to latency and determinism requirements: the end side (PLC/DCS) remains autonomous and performs setpoint tracking/lightweight control to ensure “operate-safely-when-disconnected”; the edge executes real-time inference and MV decisions under millisecond–second constraints, balancing determinism and elasticity; the cloud handles model training, production planning, and multi-factor operational optimization with batch/interactive hybrid computing. These tiering guarantees loop real-time performance with predictable delay while unlocking global optimization in the cloud.

(3) Nonintrusive Integration with PLC/DCS

Following a compatibility-first principle, the platform preserves the relative independence of existing PLC/DCS and established procedures, avoiding intrusive modifications and unnecessary coupling. Deployment adopts a progressive, expert-in-the-loop path operating in parallel with the PLC/DCS system, providing a verifiable, assessable, and controllable route for introducing new functions without changing the original system.

(4) Modular Architecture with Elastic Expansion

Functions are partitioned and decoupled into independent systems that can be deployed on demand without altering others. This design allows selecting the required system/subsystem combinations for different plants and processes, meeting real-time and security constraints while reducing integration and maintenance costs and improving cross-scenario reuse and migration.

## 4. Case Verification

### 4.1. Description of MSWI Process

**Figure 3:**
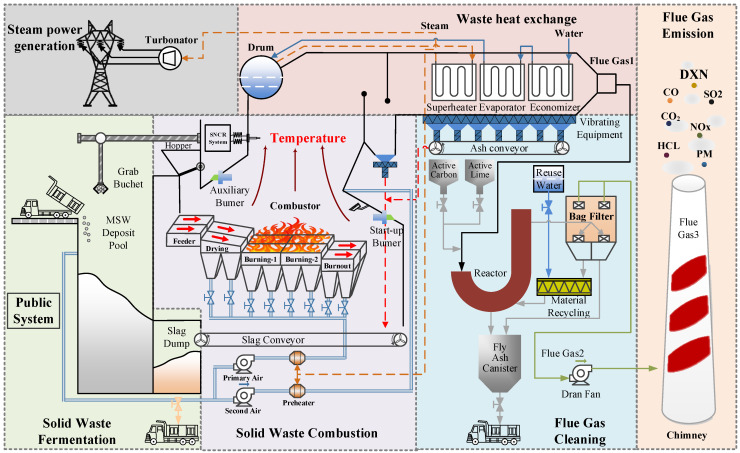
Process flow of MSWI process.

The global production of municipal solid waste (MSW) is gradually increasing [38], which has enabled the rapid development of MSWI technology with advantages such as harmlessness, reduction, and resource utilization. The process flow is shown in Figure 3.

According to Figure 3, MSW is transported by vehicles, weighed by a weighbridge, and discharged into the MSW deposit pool. After 3–7 days of biological fermentation and dehydration, the MSW is thrown into the hopper by a grab bucket. The feeder then pushes the MSW onto the grate, where it undergoes three stages: drying, combustion, and burnout. The combustion process ensures the complete decomposition and incineration of harmful substances present in the high-temperature flue gas. Typically, the flue gas temperature should be maintained above 850 °C, and the gas should stay for more than 2 s with sufficient turbulence. Subsequently, the high-temperature flue gas enters the waste heat boiler, where high-temperature steam generated from heat exchange drives the steam turbine generator unit to produce electricity. The flue gas, mixed with quicklime and activated carbon, then enters the deacidification reactor for neutralization, effectively adsorbing dioxins and heavy metals from the gas. Following this, particulate matter, neutralizing reactants, and activated carbon adsorbents are removed in the bag filter. Finally, exhaust gases containing dust, CO, NOx, SO2, HCl, HF, Hg, Cd, dioxins, and other substances are discharged into the atmosphere via chimneys. The ash generated from incineration is transported to the slag pit by a slag scraper and then transported by vehicles to designated locations for landfill or resource recovery processing.

Obviously, while performing the harmless treatment of MSW, the MSWI process utilizes the thermal energy produced by combustion to generate electricity, achieving sustainable resource development. Consequently, its primary objective is to ensure the stable combustion of MSW in the incinerator, thereby generating a stable boiler steam flow and minimizing pollutant emissions to the greatest extent possible. In developed countries, such as Japan, the automatic combustion control (ACC) system is extensively implemented to achieve stable control of MSWI processes. However, due to factors such as low calorific values, high moisture content, and inconsistent management of MSW in developing countries like China, the implementation of the ACC system faces significant challenges. At present, most MSWI plants rely on embodied intelligence to achieve stable operation, where domain experts combine their own experience, intuition, and real-time observations to make operational decisions. Although it enables effective decision-making, it often falls short of achieving true optimality. Moreover, the inherent variability in individual expertise leads to inconsistencies in operational strategies, resulting in issues such as unstable performance, fluctuating efficiency, and occasional breaches of environmental standards. Consequently, it is imperative to leverage AI technology for the operational optimization of the MSWI process. In this context, the development of the simulation platform as an engineering trial and pilot tool to validate the AI technology and software systems has emerged as an effective solution.

### 4.2. Design and Development of AISE^3^CMP-MSWI

**Figure 4:**
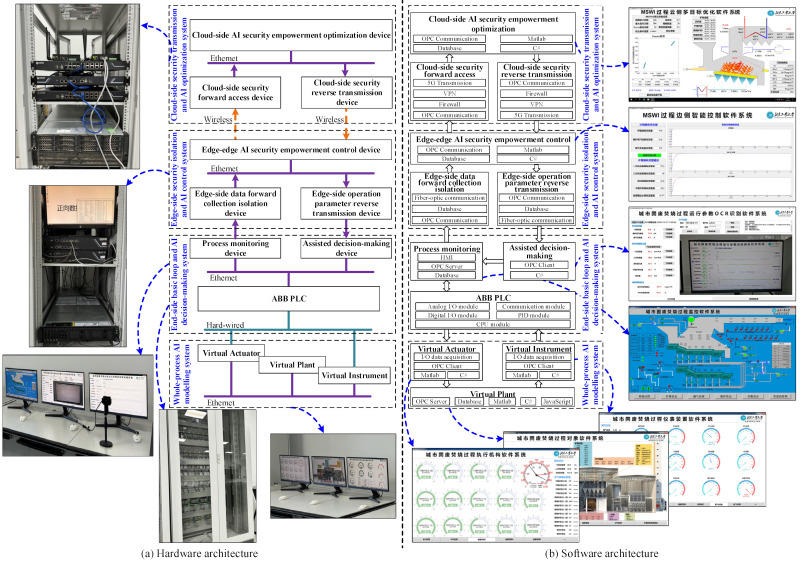
Hardware and software of AISE^3^CMP-MSWI.

The hardware and software architectures of the proposed AISE^3^CMP-MSWI are illustrated in Figure 4. In these figures, the purple solid line denotes an Ethernet connection, the blue solid line indicates a hardwired connection, and the orange dashed line represents wireless transmission. Additionally, the arrows indicate that data are transmitted unidirectionally.

As depicted in Figure 4a, from the perspective of hardware structure, the end-side basic loop is hardwired to the assisted decision-making device and the whole-process AI modeling system. Wireless data transmission occurs between edge-side AI security empowerment control device and the cloud-side security transmission and AI optimization system. Ethernet forms the basis for internal data exchange within the system.

As shown in Figure 4b, standard C#, JavaScript, and MATLAB (R2023b) are utilized for integrated programming of backend AI algorithms and frontend human–computer interaction interfaces. This resulted in the development of a software system encompassing virtual actuator, virtual plant, virtual instrumentation, process monitoring, auxiliary decision-making, edge-side AI security empowerment control, and cloud-side AI security empowerment optimization subsystems. Moreover, PLC-based loop control subsystem, edge-side forward data collection isolation, edge-side operational parameter reverse transmission, cloud-side security forward access, and cloud-side security reverse transmission subsystems are implemented using commercial hardware to ensure secure data transmission and isolation.

**Remark** **5.** *Currently, the development of AISE^3^CMP-MSWI primarily focuses on structured data transmission. Looking ahead, the goal is to incorporate AI algorithms capable of processing unstructured data, thereby further enhancing intelligence levels, integrating more data sources, and improving decision support capabilities and optimization outcomes*.

**Figure 5:**
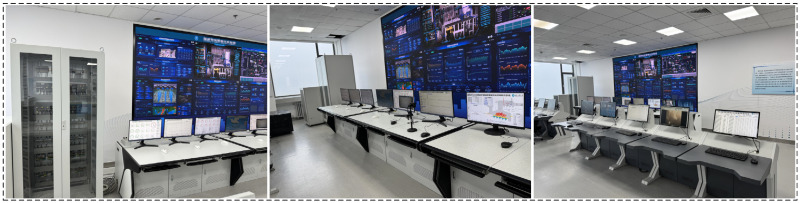
Physical diagram of AISE^3^CMP-MSWI.

The physical diagram in the laboratory environment is shown in Figure 5.

### 4.3. Verification of AISE^3^CMP-MSWI

It is important to note that the scope of this section is strictly limited to validating the functionality and design effectiveness of the developed platform. To this end, we implemented and deployed AI algorithms drawn from existing theoretical research on the MSWI process. The objective here is not the development of new or advanced AI algorithms but rather using established ones to test the platform.

#### 4.3.1. Functional Verification of End–Edge–Cloud Operation Optimization

**Figure 6:**
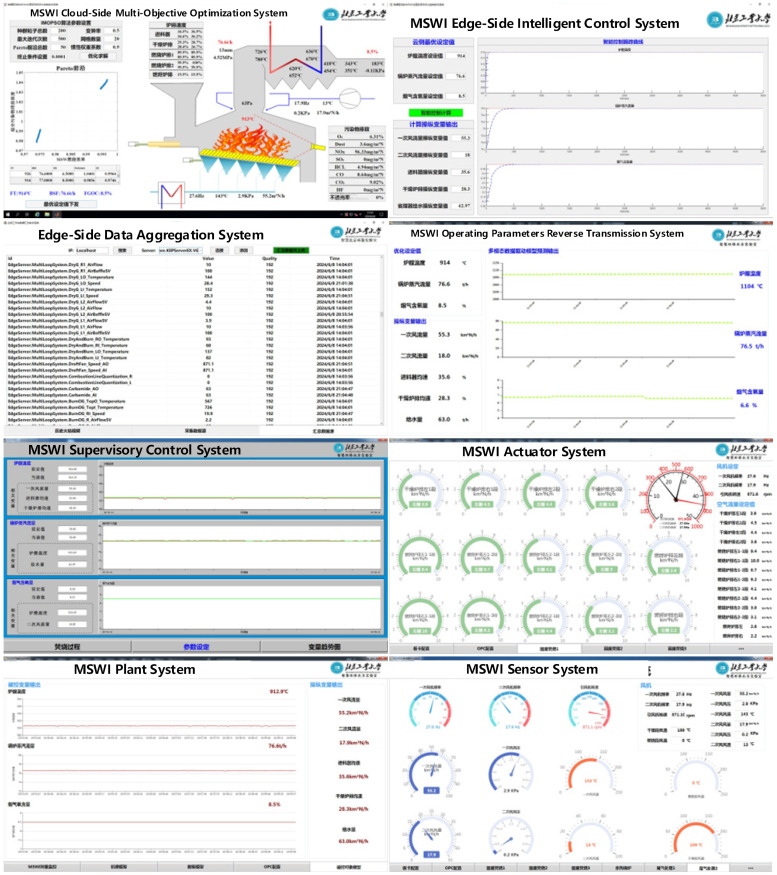
Foreground interface of AISE^3^CMP-MSWI.

To address the challenge of optimizing setpoint values of multiple controlled variables such as furnace temperature, flue gas oxygen content, and steam flow in the MSWI process, with the goal of minimizing pollutant emission concentrations and maximizing combustion efficiency, the multi-variable, multi-objective intelligent optimization control method proposed in [39] is used for programming verification. This method includes an interpretable controlled object and environmental indicator model based on an improved decision tree, a multi-loop controller algorithm based on a single-neuron adaptive PID, and an optimization algorithm using multi-objective PSO (MOPSO). The effectiveness of this method has been verified by the simulation based the actual operating data of an MSWI power plant in Beijing. Furthermore, C#, JavaScript, and MATLAB are utilized to develop the backend AI algorithms and the front-end human–computer interaction interface. The final software systems are successfully deployed in AISE^3^CMP-MSWI. The foreground interfaces are shown in Figure 6.

As shown in Figure 6, the developed AISE^3^CMP-MSWI enables smooth signal communication between various systems according to the functional design requirements. Additionally, leveraging the proposed multi-objective intelligent optimal control method, the platform verifies the accuracy of data transmission between systems and provides an effective engineering verification environment for the research of AI empowerment of the MSWI process. This platform not only offers technical support for further improving the intelligent operation of the MSWI process but also establishes a foundation for future intelligent optimization research in other CIPs.

#### 4.3.2. Verification of Modular Design and Site Transplantation

The platform proposed has modular characteristics and can be combined and built according to different requirements. The verifications of end-side AI control, edge-side AI optimization, and data isolation collection in the actual plant are shown as follows:

1. Verification of end-side AI control

**Figure 7:**
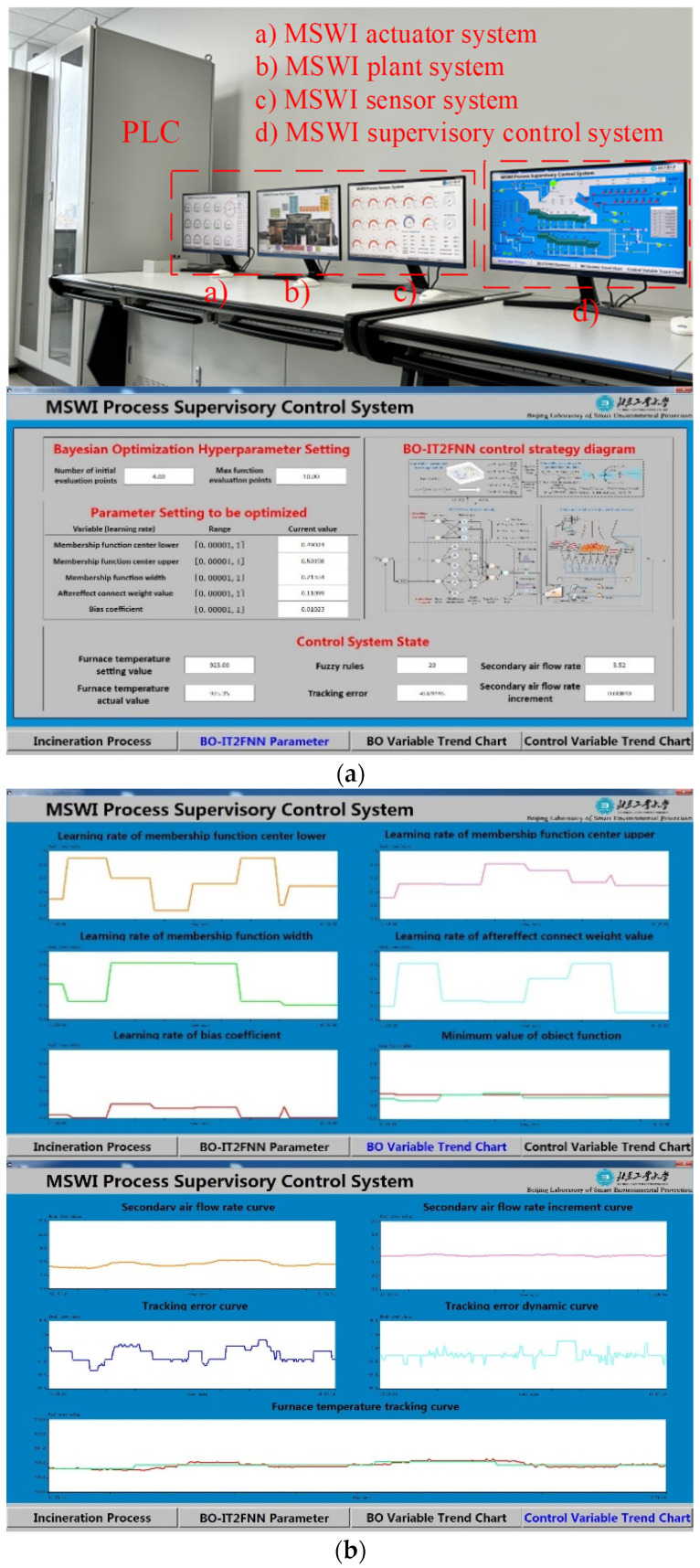
Foreground interfaces of end-side AI control. (**a**) Physical structure diagram of end-side AI control system; (**b**) foreground interfaces of process monitoring subsystem.

Furnace temperature control is crucial for ensuring the stable operation of the MSWI process and effectively reducing pollution emissions. However, traditional control strategies, such as PID used in end-side PLC/DCS devices, struggle to manage furnace temperature efficiently due to uncertainties in material composition, feeding methods, and equipment maintenance. To address this, an AI controller based on the Bayesian Optimization Interval Type-2 Fuzzy Neural Network (BO-IT2FNN) developed in [40] was employed to verify the end-side AI control. In this system, the whole-process AI modeling system for deploying the controlled object developed with an improved decision tree algorithm, the process monitoring subsystem for deploying the BO-IT2FNN algorithm in the background running mode, and the PLC-based loop control subsystem for downloading manipulated variables and uploading controlled variables within the AISE^3^CMP-MSWI were selected for validation. Figure 7 shows the foreground interfaces of the end-side AI controller.

As shown in Figure 7, a process monitoring subsystem software was developed based on the proposed BO-IT2FNN controller and successfully deployed in the AISE^3^CMP-MSWI. In Figure 7a, it is particularly noteworthy that the BO-IT2FNN controller is implemented on the monitoring computer, which transfers the manipulated variable to the PLC device via the OPC protocol for bidirectional communication. The controlled variable values, generated by the controlled object model, are fed back to the BO-IT2FNN controller through the PLC, thereby completing the closed-loop control process.

Through a user-friendly front-end interface, the software allows flexible configuration of algorithm parameters. It can continuously receive the current operating condition of the controlled object from the whole-process AI modeling system, perform real-time computation of the MV values, and feed them back to the PLC-based loop control subsystem for dynamic process adjustment. This ensures that the furnace temperature remains within the desired operating range. In addition, the process monitoring subsystem supports real-time visualization of operating condition.

2. Verification of edge-side AI optimization

**Figure 8:**
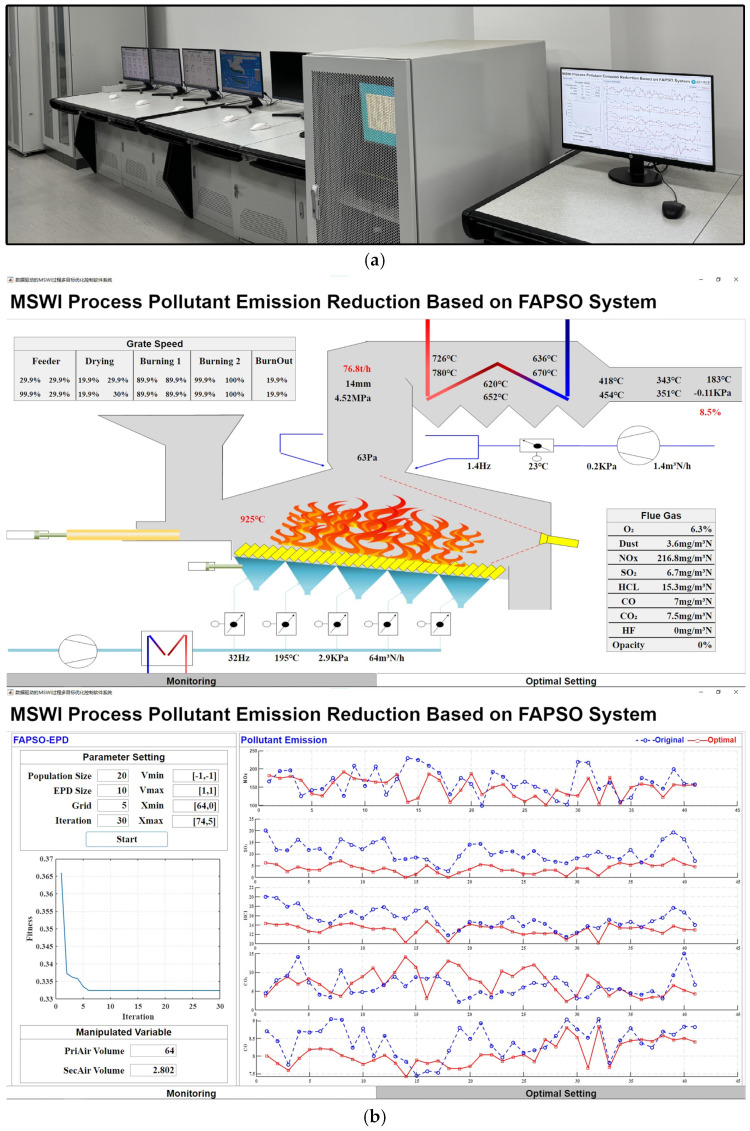

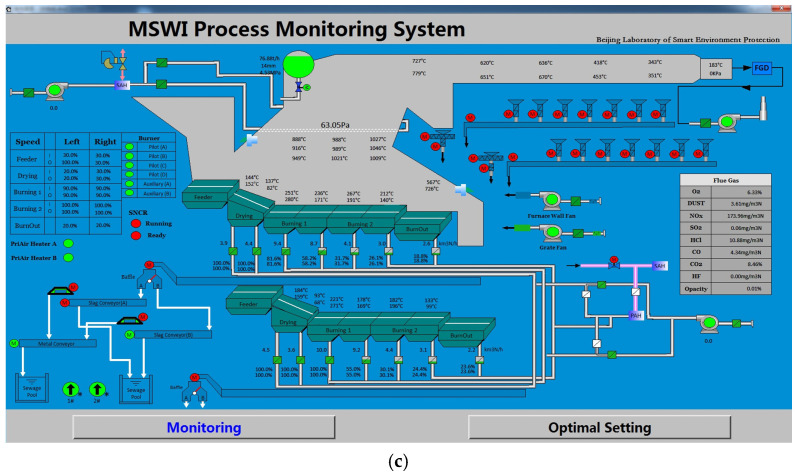
Foreground interfaces of edge-side AI algorithm. (**a**) Physical structure diagram of edge-side AI optimization; (**b**) foreground interfaces of edge-side AI optimization subsystem; (**c**) foreground interfaces of process monitoring subsystem.

To obtain the optimal “air distribution and material distribution” values in terms of minimizing pollution emissions and to replace the empirical given MV values with the manual control mode. To address this, an AI environmental indicator model based on hierarchical incremental learning for the interval type-2 fuzzy broad learning system and an AI optimization algorithm based on fuzzy adaptive particle swarm optimization (FAPSO) with an elite particle splitting (EPS) strategy, as developed in [41], were employed to verify the end-side AI control. Further, the corresponding software system was developed on end-side and edge-side systems, as illustrated in Figure 8.

As shown in Figure 8, an edge-side AI optimization subsystem software was developed based on the proposed multi-pollutant flue gas emission reduction optimization methods and successfully deployed in the AISE^3^CMP-MSWI. Similar to the process monitoring subsystem, the front-end interface allows users to customize algorithm parameters as needed while collaborating with other platform components to acquire the current operating condition and perform online computation of optimal emission reduction parameters. The experimental results demonstrate that the developed subsystem can independently compute the optimal parameters without interfering with the underlying model and transmit only the computed results to the end-side basic loop and AI-assisted decision-making system. This not only validates the effectiveness of the selected modular design but also confirms the secure isolation capability and practical value of the edge-side security isolation and AI control system.

3. Verification of data isolation collection module

**Figure 9:**
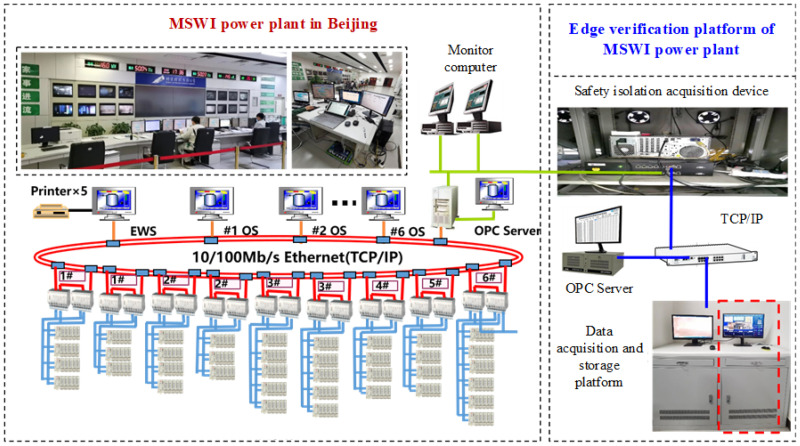
Structure of the data security acquisition system.

To facilitate in-depth research on AI algorithms for the MSWI process, we transplanted the forward data acquisition and isolation subsystem to the MSWI plant, aiming to achieve real-time acquisition and storage of industrial process data. After years of operation, the results demonstrate that the subsystem operates reliably in real-world conditions, ensuring uninterrupted operation of the Intranet system. The bidirectional protection mechanism of the subsystem guarantees secure and stable communication between the industrial site and the data processing platform, further enhancing the practical application potential of AI technology in the MSWI process. The network topology and structure of the data acquisition system are illustrated in Figure 9.

In summary, the results demonstrate that the AISE^3^CMP proposed in this article offers modular configuration capabilities, allowing for rapid assembly and construction based on actual requirements. It also serves as a convenient tool for AI algorithm validation. Moreover, although the function of different system is strictly defined, this does not negate its greater computational power and ability to handle complex tasks, which has been validated by above results.

### 4.4. Highlights and Value of AISE^3^CMP

The AISE^3^CMP-MSWI has the following highlights: (1) the first AI algorithm development and evaluation platform for the MSWI process, covering the end, edge, and cloud sides; (2) a double-layer secure isolation method is implemented to encrypt and fully verify production data and operational parameters during end-to-end data collection and transmission between the edge and cloud sides; (3) not only can multi-loop programs be developed using embedded scripts in leading DCS systems, but advanced control algorithms can also be created using high-level programming languages to enable intelligent control; (4) a comprehensive, end–edge–cloud platform is built based on open architecture and modular thinking, enhancing AI algorithm deployment efficiency and reducing secondary software development costs.

The AISE^3^CMP-MSWI has the following values: (1) Provide a platform for AI technology research and the development of innovative algorithms in research institutes: This platform focuses solely on AI modeling, control, and optimization technologies, as well as innovative algorithm research, without the need to address software or hardware architecture, thereby enhancing the efficiency and quality of output results. (2) Provide an AI technology improvement benchmark system and a pre-job training system for new employees in incineration enterprises: This platform enables the development and adaptive enhancement of AI technologies for specific furnace types and processes. By comparing with real-time data, the operational performance can be assessed, facilitating the deployment of new AI technologies and improving employee knowledge through pre-job training. (3) Provide guidance for the education of control science and engineering disciplines in industrial AI: This platform, based on a real edge–cloud system with dual-layer security isolation and virtual incineration process models, is equipped with template-based AI modeling, control, and optimization algorithms. It offers a new paradigm for industrial AI research, specifically for complex industrial processes. (4) Provide AI-powered incubators for government departments, incineration enterprises, and research institutes to collaboratively achieve an “industry-academia-research-application” objective: Focused on empowering the incineration industry through an end–edge–cloud architecture, this platform is built on AI algorithm research for the entire process, including fermentation, combustion, and purification. It facilitates the incubation of “industry-academia-research application” technologies by strengthening government supervision, addressing the growing demands of incineration enterprises, and supporting long-term exploration and research by institutes.

## 5. Conclusions

This article proposes the AISE^3^CMP platform to address the challenges in testing and validating AI algorithms for complex industrial processes on-site, as well as the difficulties in implementing end–edge–cloud architectures. The integration of end–edge–cloud architecture with a hardware-in-loop simulation platform not only enhances reliability but also provides robust support for the practical application of various AI algorithms. By clearly defining different functional modules, the platform can be adapted to meet the diverse requirements of CIP in terms of scale and type, enabling the efficient construction of a CIP platform for testing AI algorithms.

Compared to previous platforms, AISE^3^CMP—demonstrated using MSWI as an example—illustrates that AI technology can address challenges in modeling, control, optimization, and decision-making within CIP. It also enhances data transmission security through hardware with dual-layer physical isolation, while its modular design improves integration efficiency. AISE^3^CMP offers a simple and efficient modular solution for building CIP platforms, providing a streamlined path for AI implementation and serving as a key tool in advancing CIP toward end–edge–cloud collaboration and the industrial metaverse.

The limitation of this platform is that it may face challenges in adoption during implementation due to issues such as data security transmission and the protection of data privacy. The main gap in future research lies in cloud-based data collection and validating the usability of AI algorithms. Future research will focus on specific scenarios for in-depth study and gradual promotion to gain the trust of domain experts for practical application.

## Data Availability

The data presented in this study are available on request from the corresponding author.
